# Neurolymphomatosis in Recrudescent Diffuse Large B-cell Lymphoma

**DOI:** 10.22038/AOJNMB.2022.66666.1464

**Published:** 2023

**Authors:** Antonio R. Lopez, Aliyah R. Sohani, Aileen O’Shea, Thomas S.C. Ng

**Affiliations:** 1 Drexel University College of Medicine, Philadelphia, PA, USA; 2 Department of Pathology, Massachusetts General Hospital, Boston, MA, USA; 3 Department of Radiology, Division of Nuclear Medicine and Molecular Imaging, Massachusetts General Hospital, Boston, MA, USA

**Keywords:** Lymphoma, Non-Hodgkin, Neurolymphomatosis

## Abstract

Neurolymphomatosis is an uncommon manifestation of lymphoma, often presenting with painful polyneuropathy or polyradiculopathy and concomitant distal extremity weakness. Differentiation from other etiologies resulting in similar neuropathic symptoms such as compressive or inflammatory pathologies can be difficult and often results in delayed diagnosis. Here we describe a case of neurolymphomatosis affecting a 64-year-old man with a history of diffuse large B-cell lymphoma (DLBCL) in remission presenting with a right-sided foot drop following a gunshot wound. MRI at that time demonstrated thickening and enhancement of the cauda equina nerve roots. Over the course of the subsequent eight months, he developed left lower extremity sensory symptoms, left-sided foot drop and signs of upper motor neuron involvement, including left facial weakness, dysphonia, and dysphagia. ^18^F-FDG PET/CT revealed intensely avid left lumbosacral nerve roots, bilateral lower extremity and left upper extremity neurovascular bundles. Left sural nerve biopsies showed infiltration of DLBCL and confirmed neurolymphomatosis. We highlight the role of ^18^F-FDG PET/CT, with histological verification, for the diagnosis of an extended course of neurolymphomatosis occurring in the absence of typical painful neuropathy but with cranial and peripheral neuropathies.

## Introduction

 Neurolymphomatosis (NL) is a rare complication of non-Hodgkin’s lymphoma, with a majority occurring in B-cell rather than T-cell lymphoma and featuring invasion of the peripheral nervous system by malignant cells (1, 2). NL presents a diagnostic challenge as it presents similarly to other neurological complications from lymphoma including compressive neuropathy due to tumor mass, paraneoplastic neuropathy, chemoradiation toxicities. Etiologies unrelated to malignancy including inflammatory neuropathies must also be ruled out prior to diagnosing NL (2). Various methods are used to diagnose NL including positron emission tomography/computed tomography (PET/CT), magnetic resonance imaging (MRI), nerve conduction studies, cerebrospinal fluid cytology, and surgical biopsy (3). NL has previously been reported as a disease of rapid onset generally presenting with pain and asymmetric distribution of other sensorimotor symptoms (4, 5). Here we present a slowly progressing case of NL without typical radiating pain affecting the extremities or the face resulting in a patient with sensorimotor deficits affecting most limbs.

## Case Report

**Figure 1:**
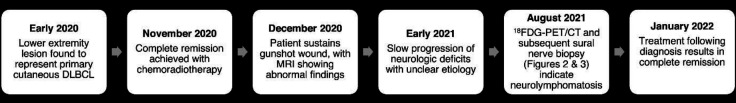
Timeline of patient’s disease and imaging course

 A 64-year-old male with a history of stage IV-A primary cutaneous diffuse large B-cell lymphoma of the left leg in remission for seven months after treatment with R-CHOP (rituximab, cyclophosphamide, doxorubicin, vincristine, prednisone) and focal radiotherapy presented with eight months of progressive facial and lower extremity weakness and loss of sensation (Figure 1). At the time of presentation for symptom evaluation, the patient had sustained a gunshot wound with a right-sided footdrop, prior to the development of additional neurological deficits, complicating evaluation. A follow up MRI lumbar spine demonstrated thickening and enhancement of the cauda equina nerve roots. 

Lumbar puncture, cytology, viral panels, and cultures were all unremarkable. Approximately one month later, the patient developed left leg numbness and then weakness. This weakness became bilateral in the ensuing weeks, resulting in bilateral foot drop. The patient also developed left facial weakness; MRI brain showed diffuse thickening and enhancement of the right facial nerve. Repeat MRI was performed approximately four months following initial lower left extremity deficits after the development of bulbar symptoms, dysphagia, left sided facial paresthesia and right sided facial weakness, which now showed bilateral facial nerve enhancement. Treatment with immunoglobulin (IVIG) resulted in no improvement and the patient subsequently developed progressive weakness in all limbs approximately 8 months after initial deficits.

**Figure 2:**
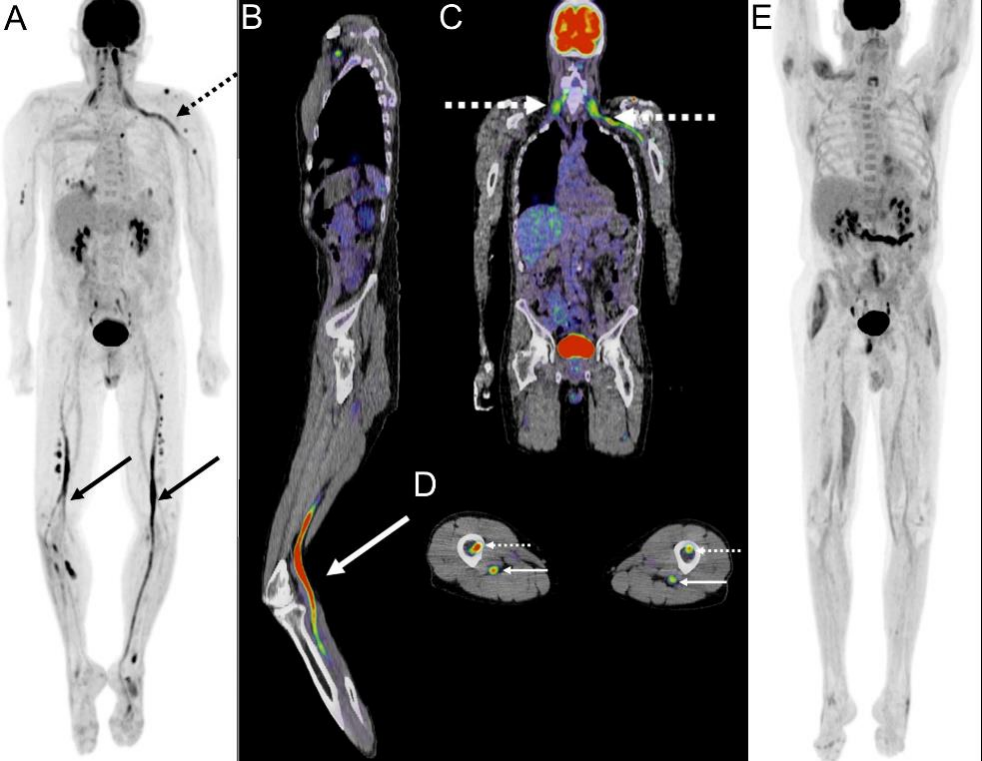
^18^F-FDG PET/CT whole-body maximum intensity projection image (**A**) shows linear abnormal uptake along the left brachial plexus (**broken arrow**), and in the bilateral sciatic nerves (**arrows**). Fused ^18^F-FDG PET/CT images demonstrate extension into the left tibial nerve (**B arrow**), involvement of the left cervicothoracic nerve roots and brachial plexus (**C, dashed arrows**), and osseus infiltration of the tibiae (**D, broken arrows**) and nearby neurovascular bundles (**D, arrows**). Follow-up ^18^F-FDG PET/CT performed 4 months later demonstrated near complete resolution at these sites (**E**). Asymmetric muscle uptake may be due to asymmetric muscular strain

^18^F-FDG/PET CT acquired at this time demonstrated abnormal foci of intense uptake in multiple sites (Figure 2). 

**Figure 3:**
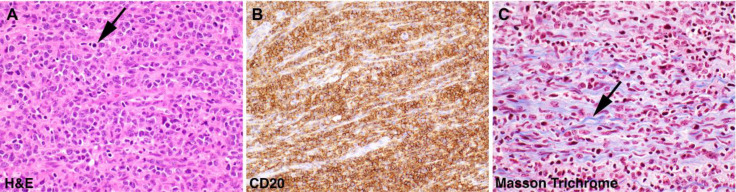
Pathologic specimens demonstrating left tibial nerve involvement by diffuse large B-cell lymphoma. **A.** The nerve was densely and diffusely infiltrated by large, atypical cells with irregular nuclei, vesicular chromatin, and prominent nucleoli. Scattered mitotic figures are present (**arrow**). **B.** Immunohistochemistry showed that the neoplastic cells were strongly positive for CD20, a marker of mature B lymphocytes. **C.** Trichrome stain highlights the damaged myelin nerve fibers in light blue (**arrow**)

 In addition to multiple sites of osseous involvement, intense uptake was seen along the left lumbosacral nerve roots, bilateral lower extremity and left upper extremity neurovascular bundles, and in the left brachial plexus. This was interpreted by the nuclear medicine physician as concerning for lymphomatous involvement, prompting biopsy. Left sural and tibial nerve biopsies showed infiltration of diffuse large B-cell lymphoma having immunophenotype and morphology compatible with relapse of the patient’s prior lymphoma (Figure 3). The patient was initially placed on high dose methotrexate and rituximab following his diagnosis, and started on rituximab, fosfamide, carboplatin, and etoposide (I-ICE) chemotherapy. He achieved full remission following an autologous stem cell transplant. 

## Discussion

 NL is the infiltration of the peripheral nervous system by lymphoma (4, 5). It is a rare neoplastic complication reported as occurring in approximately 5% of patients with lymphoma, most commonly in those with B-cell lymphoma (5, 6). Given its heterogeneous presentation and constellation of clinical findings, it can be easily confused with other etiologies including paraneoplastic and treatment-related syndromes (5, 6). MRI and ^18^F-FDG PET/CT can be useful in the diagnosis of neurolymphomatosis (7, 8). Nerves or nerve root enlargement and/or enhancement on MRI can be indicative, although not pathognomonic, for neurolymphomatosis (5). ^18^F-FDG PET/CT can be used to highlight the extent of the disease, identify sites for biopsy, and may be more sensitive for this condition earlier than other modalities (9, 10). Electrodiagnostic studies may also be used and may detect pathological changes in peripheral nerves that may be missed by imaging (11). Cerebrospinal fluid cytology is commonly used in cases of suspected NL; however, it often does not detect the presence of malignant cells (12). Histology remains the gold standard in diagnosing NL, with targeted nerve biopsies diagnostic in as high as 88% of cases (4, 13).

 Patients with NL have been reported in previous literature as presenting in one of four patterns: painful polyradiculopathy or neuropathy, cranial neuropathy, painless neuropathy, and painful or painless mononeuropathy (2, 5, 14). However, patients may have mixed presentations as has been reported in the past, and as in the case of our patient who presents with slowly progressive painless cranial neuropathy, radiculopathy, and mononeuropathy that has not previously been reported in the literature (15). The cauda equine nerves, the brachial plexus, and the sciatic nerve are among the most commonly affected peripheral targets for NL (30-35%) and the tibial and sural nerve is among the least common (2.5%) (12). Most NL cases spare the cranial nerves and result in sensorimotor deficits with pain but without autonomic abnormalities (12). The time course for NL is variable, with slowly progressing NL occurring over many months, as seen in our patient, and more rapidly progressing NL occurring in a subacute time frame having both been previously reported (16). The mainstay of treatment for neurolymphomatosis is systemic chemotherapy and includes various regimens such as CHOP (cyclophosphamide, doxorubicin, vincristine, prednisone), MCHOD (methotrexate, cyclophosphamide, doxorubicin, vincristine, dexamethasone) and others without a clear indication of superiority among the regimens (17). While this may provide symptomatic relief and decrease lymphomatous burden, responses are variable, and relapse is common – with median survival as low as 10 months and only 24% of patients surviving three years after diagnosis (4, 18). Radiation therapy may be used alongside systemic chemotherapy with positive results in localized disease (17).

 Our patient provided a puzzling, extended course involving clinical features that appeared after incidental findings via MRI taken in the setting of a gunshot wound and that matched a combination of standard patterns that previous literature has used to categorize NL including cranial neuropathy, radiculopathy, and mononeuropathy all without the typical feature of pain. Such a pattern of slowly progressive symptoms in the context of painless neuropathy has, to our knowledge, not been described previously. In our patient, ^18^F-FDG PET/CT showed infiltration of common locations such as the sciatic nerves but also uncommon locations such as the tibial nerve and the cervicothoracic nerve roots. NL should be considered in patients with a history of lymphoma suffering from sensorimotor symptoms that affect that feature multiple limbs even in cases without rapid progression and/or painful polyradiculopathy and polyneuropathy. 
